# The rare *BRAF VK600-601E* mutation as a possible indicator of poor prognosis in rectal carcinoma – a report of a case

**DOI:** 10.1186/s12881-015-0144-7

**Published:** 2015-01-31

**Authors:** Yoshiko Mori, Takeshi Nagasaka, Hideyuki Mishima, Yuzo Umeda, Ryo Inada, Hiroyuki Kishimoto, Ajay Goel, Toshiyoshi Fujiwara

**Affiliations:** Department of Gastroenterological Surgery, Okayama University Graduate School of Medicine Dentistry and Pharmaceutical Sciences, 2-5-1 Shikata-cho, Kita-ku, Okayama 700-8558 Japan; Cancer Center, Aichi Medical University, 1-1 Yazakokarimata, Nagakute City, Aichi 480-1195 Japan; Center of Gastrointestinal Research; and Center for Epigenomics, Cancer Prevention and Cancer Genomics, Baylor Research Institute and Charles A. Sammons Cancer Center, Baylor University Medical Center, 3500 Gaston Avenue, Suite H-250, Dallas, TX 75246 USA

**Keywords:** Rectal cancer, *BRAF* mutation, *BRAF* VK600-601E, Chemotherapy, Prognosis

## Abstract

**Background:**

The *BRAF* V600E mutation is reportedly associated with inferior survival among colon cancer patients. Here we report a patient with rectal cancer who carried the novel *BRAF* mutation VK600–601E, which has analogous molecular functions to those of the conventional *BRAF* mutation *V600E*, and may have potential as a prognostic marker for colorectal cancer (CRC).

**Case presentation:**

The present 65-year-old male patient was diagnosed with recurrent rectal adenocarcinoma (stage II by AJCC TNM staging 7th edition) 14 months after surgery and was treated with modified FOLFOX6 (fluorouracil, leucovorin, and oxaliplatin), radiation, and FOLFIRI (fluorouracil, leucovorin, and irinotecan). The tumor progressed before further treatment could be initiated, resulting in death after 15 months. This survival period was similar to the median overall survival among patients with metastatic CRC and *BRAF* mutations who were treated with the FOLFIRI regimen with or without cetuximab.

**Conclusions:**

Thus, the *BRAF* VK600–601E mutation may lead to an aggressive clinical course in CRC patients suffering from rapid progression and potential resistance to multiple therapeutic modalities.

## Background

Prognoses for patients with colorectal cancer (CRC) have improved significantly with the introduction of molecular-targeted drugs such as anti-epidermal growth factor receptor agents (anti-EGFR). First-line treatment with the anti-EGFR cetuximab in addition to FOLFIRI (fluorouracil, leucovorin, and irinotecan) reduced the risk of progression of metastatic colorectal cancer compared with FOLFIRI treatment alone. However, the benefit of cetuximab was limited to patients with *KRAS* (codon 12 and 13) wild-type tumors [1]. S Tejpar et al. recently reported a number of candidate markers that influence the response to anti-EGFR, even among patients with wild type *KRAS*, including new *KRAS* mutations (codon 61 and 146) and mutations in *BRAF* and *NRAS* [2]. Although the best characterized *KRAS* mutations on codons 12 and 13 are important, other *KRAS* mutations, such as those on codons 61 and 146, have received recent attention.

BRAF is a member of the RAF family of kinases and operates by binding to RAS [3]. A recent retrospective study of several clinical trials demonstrated that the presence of the *BRAF* V600E mutation was a strong prognostic factor for overall survival (OS) in patients with stage II/III CRC, particularly for tumors with low or stable microsatellite instability (MSI-Low, MSI-Stable, or no MSI) [4]. Activating mutations in the *BRAF* gene are almost within the kinase domain and produce a signaling substitution of valine for glutamic acid at position 600 (V600E) [5]. However, in the present rectal cancer patient with wild-type *KRAS* and no MSI, we discovered a novel *BRAF* mutation that led to a triplet deletion of the coding nucleotides 1799–1801 (TGA1799–1801 deletion; VK600–601E). This patient demonstrated relatively poor responses to conventional chemotherapy. Although this mutation has only been found in one patient to date, testing for this and other novel *RAS* mutations (*KRAS* codons 61 and 146 or *NRAS*) may provide essential prognostic markers that can be used to individualize treatment regimens for CRC patients.

## Case presentation

**Figure 1:**
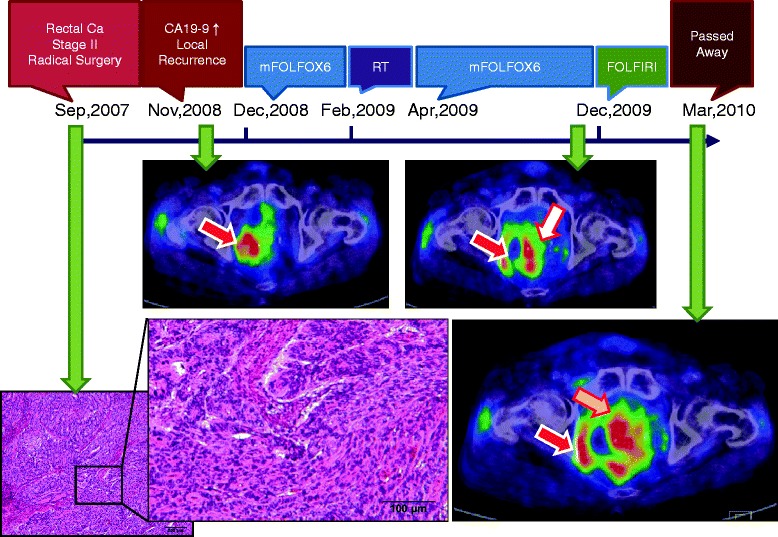
**Timeline of a rectal cancer patient with the** ***BRAF VK600–601E*** **mutation.** The left lower image shows the findings of the histopathological examination of the resected tumor following initial surgery in 2007, revealing a moderately to poorly differentiated adenocarcinoma. PET/CT slices show recurrent tumors; progressive perineal metastatic tumor (the white arrow) and right obturator and right internal iliac lymph node metastases (the red arrows). The tumors had infiltrated into the right internal iliac artery and right urinary duct. The perineal metastatic tumor was enlarged and had invaded the bladder (yellow arrow).

A 65-year-old man presented with perineal pain, pollakiuria, and a serum carbohydrate antigen 19–9 level of 564.3 IU/mL (normal, <39.9 IU/mL). He had undergone a resection without preoperative chemoradiotherapy for a 4 × 4.5-cm rectal adenocarcinoma lesion 14 months previously, which was graded as IIA (T3N0M0) according to the AJCC TNM classification [6]. According to the surgical report, abdominoperineal resection with lateral lymph nodes resection and inferior mesenteric artery lymph node resection had done. Computed tomography (CT) and positron emission tomography (PET)/CT revealed a 3.5 × 3.0 × 2.7-cm perineal metastatic lesion obstructing the right ureter. Metastases were observed in the right obturator lymph node, and the largest lesion measured 4.0 × 3.3 × 3.0 cm. The metastatic lymph node had infiltrated the right internal iliac artery (Figure 1).

After stent placement in the ureter, four cycles of a modified FOLFOX6 regimen (mFOLFOX6) comprising 85 mg/m^2^ oxaliplatin, 200 mg/m^2^ leucovorin, 400 mg/m^2^ 5-fluorouracil (5FU) bolus on day 1 and 2400 mg/m^2^ 5FU as a 46-h continuous infusion were administered at 2-week intervals. Subsequently, the patient experienced lower back pain, and a second PET/CT examination indicated tumor recurrence and lymph node metastases on one side of the pelvis. Chemotherapy was terminated and radiotherapy (30 fractions at 2 Gy per day; total, 60 Gy) was initiated. Upon pain relief, mFOLFOX6 therapy was reinitiated according to the previous regimen, and no signs of disease progression were observed until 10 months later. At this point PET/CT scans revealed that the perineal metastatic tumor had enlarged to approximately 9 cm (Figure 1), and the patient was hospitalized and treated with a FOLFIRI regimen comprising 150 mg/m^2^ irinotecan, 200 mg/m^2^ leucovorin, 400 mg/m^2^ 5FU bolus on day 1 and 2400 mg/m^2^ 5FU as a 46-h continuous infusion. One week later, the patient developed grade 4 neutropenia (<500/mm^3^; Common Terminology Criteria for Adverse Events ver. 4.0).

**Figure 2:**
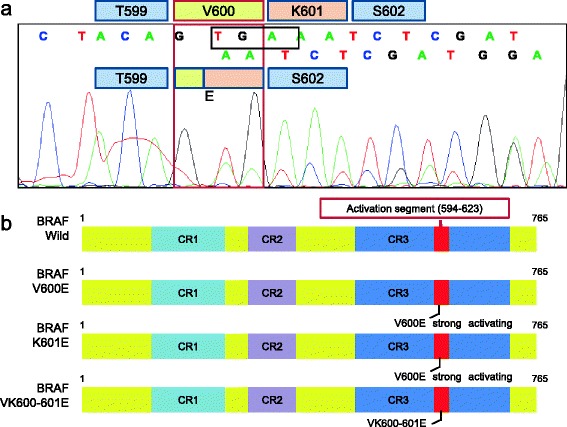
***BRAF*** **mutation analysis of the primary tumor. (a)** Direct sequencing chromatographs of the *BRAF VK600–601E* mutation demonstrating triplet deletion from T1799 to A1801. **(b)** Schematic representation of the BRAF protein structure and various mutations. CR-1, −2, and −3 represent conserved regions. The kinase activation segment is located between codons 594 and 623. Modified from Wan PT et al. [12].

Between treatments, DNA was extracted from sections of the primary tumor tissue and analyzed for *KRAS* and *BRAF* mutations and MSI [7]. No MSI or mutations at codons 12 or 13 of *KRAS* were identified. Direct sequencing for *BRAF* revealed a triplet nucleotide deletion (TGA) in coding nucleotides 1799–1801 (Figure 2). This mutation resulted in the deletion of amino acid 601 (lysine) and a valine–glutamate substitution at position 600 (VK600–601E).

After recovery from severe neutropenia, the perineal metastatic tumor had grown rapidly and had invaded the bladder (Figure 1), and consequent severe hematuria and nephropyelitis resulted in renal failure. Despite therapy for renal failure, the patient died 15 months after initiation of first-line therapy for tumor recurrence.

## Conclusions

CRC development is considered a multistep process that follows the accumulation of genetic alterations, including chromosomal abnormalities, gene mutations, and epigenetic changes [4]. The Ras–Raf–MAP kinase pathway is known to mediate cellular responses to extracellular signals that regulate cell proliferation, differentiation, and apoptosis [8]. *KRAS*-activating mutations decrease or abolish the intrinsic GTPase activity of the KRAS protein, leading to its constitutive activation. Similarly, the *BRAF* V600E mutation induces structural changes that increase the kinase activity of the RAF protein [9]. Moreover, tumors with *RAS* oncogene mutations are resistant to treatments with EGFR inhibitors, indicating that mutations in the *RAS* proto-oncogene are predictive of treatment responses [4,10]. *RAS* mutations commonly occur in codons 12 and 13 and are implicated in many human cancers, including approximately 40% of CRC cases [3]. In 2002, Davies et al. [5] identified activating mutations in *BRAF* that were present in many human cancers, including approximately 10% of CRC cases. The *BRAF* V600E mutation accounts for 80% of *BRAF* mutations in human cancers and is thought to be biologically distinct from less frequent *BRAF* mutations because it allows growth in the absence of functional RAS genes [5]. Interestingly, the *BRAF* V600E mutation has not been previously reported in combination with the *KRAS* mutation in patients with CRC [3,11], suggesting that at least one of these pathways must remain intact for cell survival.

The *BRAF* V600E mutation is known to be a strong prognostic marker in patients with metastatic and stage II/III CRC [4]. Similar to most activating mutations, the *BRAF* VK600–601E mutation affects the activation segment of BRAF. Therefore, these mutations likely disrupt the interaction between the activation segment and P-loops that normally stabilize BRAF in the inactive conformation [12,13]. Because the *BRAF* VK600–601E mutation is functionally analogous to the *BRAF* V600E mutation [14], it may be an additional marker of recurrence and poor treatment responses in patients with CRC. In support of this hypothesis, the present CRC patient responded poorly to conventional radiotherapy and chemotherapy and died at 15 months after initiation of first-line treatment for recurrence. This time period was almost equal to the median overall survival of patients with metastatic CRC with *BRAF* mutations who were treated with the FOLFIRI regimen with or without cetuximab (14.0 and 10.3 months, respectively) [15]. Although outcomes of rectal cancer often differ from those of colon cancer, clinical trials for unresectable advanced colorectal cancer always include colon and rectal cancer cases. For example, in a randomized phase III study (CRYSTAL trial) comparing FOLFIRI alone with cetuximab plus FOLFIRI regimens in 348 patients with *KRAS* exon2 wild-type CRCs, rectal cancer cases comprised 40.9% and 44.2% of the treatment arms, respectively [1].

To our knowledge, this is the first report describing the *BRAF* VK600–601E mutation in a patient with CRC. Although this mutation may be uncommon, the present observations warrant routine investigation of alternative mutations among CRC patients who show rapid progression and/or resistance to aggressive radiotherapy and/or chemotherapy.

## Consent

This is a report of a case of standard treatment of the CRC with the rare somatic mutation. Written informed consent was obtained from the patient for testing the mutation status. Written informed consent for publication of this case report and accompanying images was obtained from a kin of the patient. However, we had not presented to our ethics committee, because this is not interventional study.
